# Supplementary material for: Socioeconomic Disparities in Diet Vary According to Migration Status among Adolescents in Belgium
Source: Nutrients. 2019 Apr 10;11(4):812. doi: 10.3390/nu11040812 (PMC6520743; doi:10.3390/nu11040812)
Supplement: Supplementary file 1 [file nutrients-11-00812-s001.zip › TableS8.docx]

**Supplementary Table 8.** Comparison of food consumptions, cultural and sociodemographic characteristics between eligible participants included in the analyses and those excluded due to missing data — HBSC, Belgium, 2014

|  | **Included participants (*n* = 19,172)** | | **Eligible participants not included (*n_max_* = 3859)** | |  |
| --- | --- | --- | --- | --- | --- |
|  |  |  |  |  |  |
|  | ***n*** | **%** | ***n*** | **%** | ***P* value** |
| **Fruit** |  |  | *3634* |  | 0.004 |
| >once a day |  | 17.2 |  | 19.4 |  |
| 5–7 days a week |  | 32.8 |  | 31.2 |  |
| <5 days a week |  | 50.0 |  | 49.4 |  |
| **Vegetable** | *18,974* |  | *3674* |  | <0.001 |
| >once a day |  | 18.8 |  | 19.9 |  |
| 5–7 days a week |  | 55.6 |  | 48.7 |  |
| <5 days a week |  | 25.6 |  | 31.4 |  |
| **Fish** | *18,924* |  | *3676* |  | <0.001 |
| ≥two days a week |  | 20.2 |  | 24.3 |  |
| <two days a week |  | 79.8 |  | 75.7 |  |
| **Dairy products** | *18,541* |  | *3539* |  | 0.20 |
| >once a day |  | 79.6 |  | 80.6 |  |
| ≤once a day |  | 20.4 |  | 19.4 |  |
| **Chips and fries** | *18,853* |  | *3615* |  | <0.001 |
| <once a day |  | 87.7 |  | 81.1 |  |
| ≥once a day |  | 12.2 |  | 18.9 |  |
| **Sugar-sweetened beverages** | *18,642* |  | *3567* |  | <0.001 |
| ≤once a week |  | 24.5 |  | 21.3 |  |
| 2–6 days a week |  | 31.8 |  | 29.9 |  |
| ≥once a day |  | 43.7 |  | 48.8 |  |
| **Gender** |  |  | *3859* |  | <0.001 |
| Boys |  | 50.6 |  | 58.9 |  |
| Girls |  | 49.4 |  | 41.1 |  |
| **Age** |  |  | *3859* |  | <0.001 |
| 10–12 years |  | 28.8 |  | 31.8 |  |
| 13–16 years |  | 50.2 |  | 47.1 |  |
| 17–19 years |  | 21.0 |  | 21.1 |  |
| **Migration status** **^a^** |  |  | *3381* |  | <0.001 |
| Natives |  | 69.6 |  | 59.8 |  |
| 2nd-generation immigrants |  | 22.0 |  | 25.8 |  |
| 1st-generation immigrants |  | 8.4 |  | 14.4 |  |
| **Family structure ^a^** |  |  | *2955* |  | <0.001 |
| Two parents |  | 66.4 |  | 58.4 |  |
| Blended family |  | 14.1 |  | 15.6 |  |
| Single-parent family |  | 19.5 |  | 26.0 |  |
| **Family Affluence Scale ^a^** |  |  | *2089* |  | <0.001 |
| High |  | 19.4 |  | 15.4 |  |
| Medium |  | 63.7 |  | 56.7 |  |
| Low |  | 16.9 |  | 27.9 |  |
| **Parental working status ^a^** |  |  | *3314* |  | <0.001 |
| Both parents working |  | 68.4 |  | 52.7 |  |
| One working, the other at home |  | 17.4 |  | 16.6 |  |
| One working, the other not at home |  | 8.1 |  | 12.2 |  |
| None working |  | 6.1 |  | 18.5 |  |
| **Siblings** |  |  | *2606* |  | 0.52 |
| Single child |  | 9.3 |  | 9.7 |  |
| Siblings |  | 90.7 |  | 90.3 |  |
| **School Region** |  |  | *3859* |  | <0.001 |
| Brussels-Capital Region |  | 11.4 |  | 15.2 |  |
| Walloon Region |  | 46.6 |  | 49.4 |  |
| Flemish Region |  | 42.0 |  | 35.4 |  |

^a^ For details, see *Methods* section
